# Metalloproteomics: principles, challenges and applications to neurodegeneration

**DOI:** 10.3389/fnagi.2013.00035

**Published:** 2013-07-18

**Authors:** Amber Lothian, Dominic J. Hare, Rudolf Grimm, Timothy M. Ryan, Colin L. Masters, Blaine R. Roberts

**Affiliations:** ^1^The Florey Institute of Neuroscience and Mental Health, The University of MelbourneParkville, VIC, Australia; ^2^Elemental Bio-Imaging Facility, University of Technology SydneyBroadway, NSW, Australia; ^3^Life Sciences Group, Agilent TechnologiesSanta Clara, CA, USA

**Keywords:** metalloproteomics, LC-ICP-MS, metals in neurodegeneration, quantitative methods, hyphenated ICP-MS techniques

## Abstract

Trace elements are required for a variety of normal biological functions. As our understanding of neurodegenerative disease advances we are identifying a number of metalloenzymes involved in disease process. Thus, the future of metals in neurobiology will rely more on detailed information regarding what metalloenzymes are present and how they are involved in the pathophysiology of disease. To gain this detailed information, we will rely less on bulk measures of the amount of a trace elements in a particular tissue and turn to metalloproteomic techniques to help elucidate both metalloprotein structure *and* function. Recent advances in metalloproteomics will translate to a richer understanding of the mechanism and precise role of metalloenzymes and proteins in the brain.

## INTRODUCTION

The brain is an incredibly complex organ that has an equally unique metabolic need. In humans, although the brain only composes about 2% of body mass, it consumes approximately 25% of the energy output. The excessive proportion of metabolic activity occurring in the brain, compared to the whole body is seen in numerous examples of metal-mediated cellular function. Metalloenzymes are important for all aspects of physiology, including mitochondrial function, transcriptional regulation, catabolism, and, for the brain, the production of the important secondary messenger nitric oxide (NO) by NO synthase, which depends on Fe and Zn (Mayer et al., 1991; Li et al., 1999). In line with the brain’s tendency for excess, the production of NO is about 20 times greater for the central nervous system compared to the vasculature (Salter et al., 1991; Garthwaite and Boulton, 1995; Pacher et al., 2007). Further, the actual signaling pathway for NO is dependent on Fe bound to a heme in soluble guanylate cyclase (Gerzer et al., 1981; Ignarro et al., 1982). The role of metals in NO production and signaling is just one example of the vital role trace elements have in biology. Indeed, life itself would not exist without oxygen produced by chloroplasts, and transport through our body by hemoglobin, both of which require a metal ion for function.

Despite the intense amount of research into cellular mechanisms metalloenzymes have largely been overlooked, yet the “metalloproteome” dictates much of the reactivity within a cell. The targeted investigation of metalloproteins in the central nervous system will provide mechanistic insights into how reported changes in total levels of trace elements translates to specific proteins.

## WHAT IS “METALLOPROTEOMICS”?

Over a decade ago, Glen Evans commented on the “omic” science revolution, stating that the advent of post-genomic sciences was brought about by a need to “analyze the components of a living organism in its entirety” (Evans, 2000). As systems biology has become more integrated into the modern laboratory the traditional streams of “omic” sciences have diversified to include specific fields of study examining the functional components of biomolecules, rather than simply their presence or structure.

Metalloproteomics is one such newly established area of study, which amalgamates proteomic and metallomic approaches to biology (Barnett et al., 2012). Proteomics is the large-scale investigation of the structural and functional properties of proteins (Anderson and Anderson, 1998), whereas metallomics encompasses the “comprehensive analysis of the entirety of metal and metalloid species within a cell or tissue type” (Szpunar, 2005). We believe the term “metalloproteomics” is more suited to the field than metallomics alone, as it recognizes the important relationship between biometals and proteins, rather than focusing solely on the presence of an individual metal species. In 2004, Hiroki Haraguchi first described metallomics as “integrated biometal science” (Haraguchi, 2004), though it is only in recent years that the potential of integrating high-end atomic spectrometry techniques into typical proteomics workflows is beginning to gain attention.

It is estimated that around one-third of all proteins in the human body require a metal cofactor for functionality (Andreini et al., 2008; Waldron et al., 2009; Barnett et al., 2012). Redox properties of metal ions mediate a plethora of cellular processes, from the electron transport mechanisms within mitochondria to the formation of myelin in developing nerve cells. Metals have the ability to interact with multiple proteins, all with varying functions, located in every cell of the human body. Due to the abundance of proteins that are estimated to require a metal cofactor for function, this is an area that requires extensive work to be done in order to characterize the vital role metals may play in the molecular basis of disease.

Unlike glycosylation and phosphorylation, which do not always have a one-to-one relationship with protein function (Jensen, 2006), the presence of a metal cofactor is intimately linked with enzymatic function. For example the function of Cu,ZnSOD is dependent on the presence of both metals. The Zn-only containing enzyme does occur in transgenic animal models overexpressing the enzyme (Lelie et al., 2011; Rhoads et al., 2011), though it does not pose any superoxide scavenging ability without Cu. SOD can even produce superoxide rather than scavenge it in the absence of Zn (Estévez et al., 1999). Bottom-up proteomics neglect information on non-covalent cofactors, including metals. The overall goal of systems biology or proteomics is to measure how proteins change to help elucidate function, hence the interest in glycosylation and phosphorylation and other post-translational modifications. However, the functional importance of non-covalent cofactors has the promise to determine the functional output of proteins, and cannot be overlooked.

## HOW BIG IS THE “METALLOPROTEOME”?

The proportion of metalloproteins in the proteome is still widely unknown. Even in relatively simple single-cellular organisms, the number of metalloproteins that have been comprehensively identified is only a fraction of the one-third of all proteins predicted to bind metals, most likely due to technical limitations. This suggests that studying the human metalloproteome will encounter significant difficulties, as the number of metalloproteins that it will encode for is larger then that of relatively simple prokaryotes.

Relatively few research groups are applying new technologies to metalloproteomics. One such group that has been actively conducting research in this field and has confirmed the lack of characterization of metalloproteins is that lead by John Tainer and Michael Adams. Their recent study of metalloproteins in *Pyrococcus furiosus* illustrates the difficulties involved in identifying metalloproteins found in even the most basic of life forms (Cvetkovic et al., 2010). It is estimated that there are around 2,000 encoded proteins in the *P. furiosus* genome (Lee et al., 2009), and if, as stated previously, one-third of them are expected to be metalloproteins, around 600 metalloproteins should be present. However, experiments conducted by the Tainer and Adams group demonstrated that only 50% of the metal peaks they analyzed contained a protein that could be linked to a known metalloprotein (Cvetkovic et al., 2010). This study demonstrates the large gap in our knowledge of what proteins even utilize a metal ion.

**FIGURE 1:**
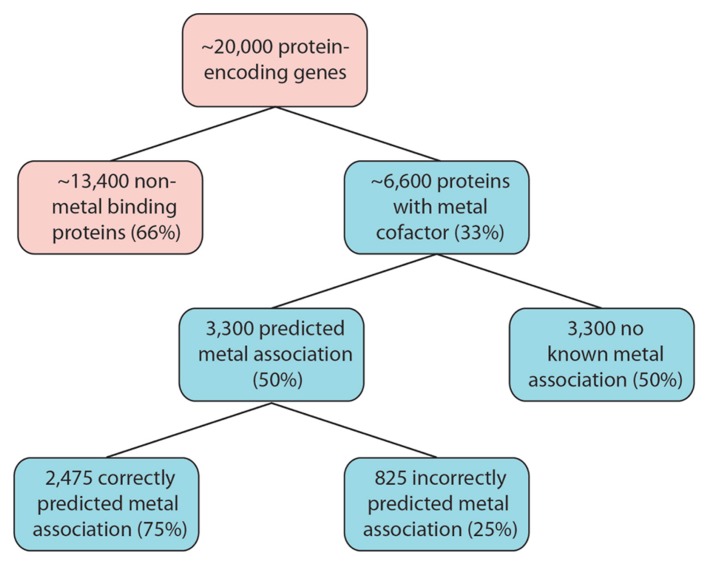
** Estimated size of the metalloproteome.** Following the example of *P. furiosus*, as many as 6,600 human proteins are estimated to have metal association. Our limited knowledge of metalloproteins, combined with analytical limitations, will greatly impact on our ability to correctly predict the metal association, and therefore function, as a large number of these proteins are identified.

A conservative interpretation of these results is that for any organism yet to have it’s metalloproteome mapped, 50% of these metalloproteins will have the metal association incorrectly predicted or it will not yet be known. In the human proteome, which consists of around 20,000 protein-encoding genes, an estimated number of unknown or misidentified metalloproteins can be predicted. About 6,600 protein-encoding genes will encode for metalloproteins (Waldron et al., 2009). Using *P. furiosus *as a guide, 3,300 of these will have a predicted metal association, of which a further three-quarter will exhibit a correctly predicted metal association. This leaves an estimated 4,125 metalloprotein-encoding genes that will have an incorrect metal association predicted, by current bioinformatic tools, or display no metal interactions (**Figure 1**). The complexity of a multi-cellular complex organism like the human body is expected to have a wider range of metal–protein interactions than those observed in *P. furiosus.* It should, however, serve as an indication of the complex task at hand that faces scientists embarking on the next phase of systems biology that encompasses this functional component of an organism.

## WHAT ARE THE ANALYTICAL CHALLENGES TO METALLOPROTEOMICS?

The central issue that hampers the characterization of metalloproteins is difficulty in preserving their native state during analysis. Traditional proteomic approaches are generally incompatible for studying metal–protein interactions, as they tend to require denaturing conditions and enzymatic digestion, leading to disruption of the comparatively weak ionic interactions governing most metalloprotein bonds. A targeted metalloproteomics approach that acknowledges the importance of retaining native conditions is the answer to these issues, as it will provide the capability to determine the roles that metals play in the functional properties of proteins in biological systems, whilst ensuring that the detailed structural analysis of proteins is still obtainable.

The central tenet to characterizing metalloproteins is that in order to correctly identify a species the metal must still be bound to the protein. Once metalloproteins are no longer in their native state, misincorporation of metals or complete loss of metal becomes a significant problem. To accurately quantify metalloproteins it is vital that their native state is kept intact and is not altered by denaturing conditions. The use of strong acids/bases, concentrated inorganic salt, organic solvents and heat all contribute to the loss of native folded states. Thus, chromatographic separation for metalloproteomics should endeavor to use buffers that are of physiological pH, as this will help to prevent alterations to the secondary and tertiary structures that lead to the loss of metal binding. A metalloproteomics workflow must ensure that each possible source of experimental error is mitigated to a point where its influence is negligible.

Experimental error may be encountered even prior to analysis at the point of sampling. Metalloprotein integrity may be disrupted by reagents and buffers used in collection or sample preparation, and even storage conditions (Manley et al., 2009) such as repeated freeze-thaw cycles, that have an uncharacterized effect on metal–protein interactions. The post-mortem stability of SOD shows that the time in which it takes to freeze the sample post-collection does not have any significant effect on the concentration of the protein (Brooksbank and Balazs, 1984). However, the post-mortem stability of other metalloproteins is an area for further investigation. Error during this initial step of the experimentation causing loss of bound metals will impact all of the conclusions that are drawn from the experiment. Even relatively inert chemicals, such as acetate buffers used in native size-exclusion chromatography (SEC), may impart unwanted effects on metal binding through the presence of a relatively strong complexing anion (Inagaki et al., 2000; Wang et al., 2007; El Balkhi et al., 2010).

Characterizing an unknown metalloprotein within an environment that is rich in proteins is exceptionally challenging. Metalloproteomic techniques have been used to characterize known metalloproteins, such as metallothionein isoforms (Chassaigne and Lobiński, 1998). Targeted proteomic approaches where the specific nature of the protein in question is already known does not present as many difficulties as a *de novo* approach, since these proteins can be targeted for isolation. There are multiple ways protein can be targeted and measured using a combination of chromatography techniques (del Castillo Busto et al., 2005) coupled to inductively coupled plasma-mass spectrometry (ICP-MS) and more recently with mass spectrometry. For example, mass spectrometry including top-down techniques have been used to characterize both apo- and metallated-metallothionein in both rabbit and dolphin liver as well as horse kidney (Chassaigne and Lobinski, 1998; Chassaigne and Lobiński, 1998; Ryvolova et al., 2011; Pedrero et al., 2012). These studies highlight the kind of detailed information that can be obtained by using a combination of mass spectrometry and chromatography to study a particular protein.

## HOW DO WE OVERCOME THESE ANALYTICAL LIMITATIONS?

**FIGURE 2:**
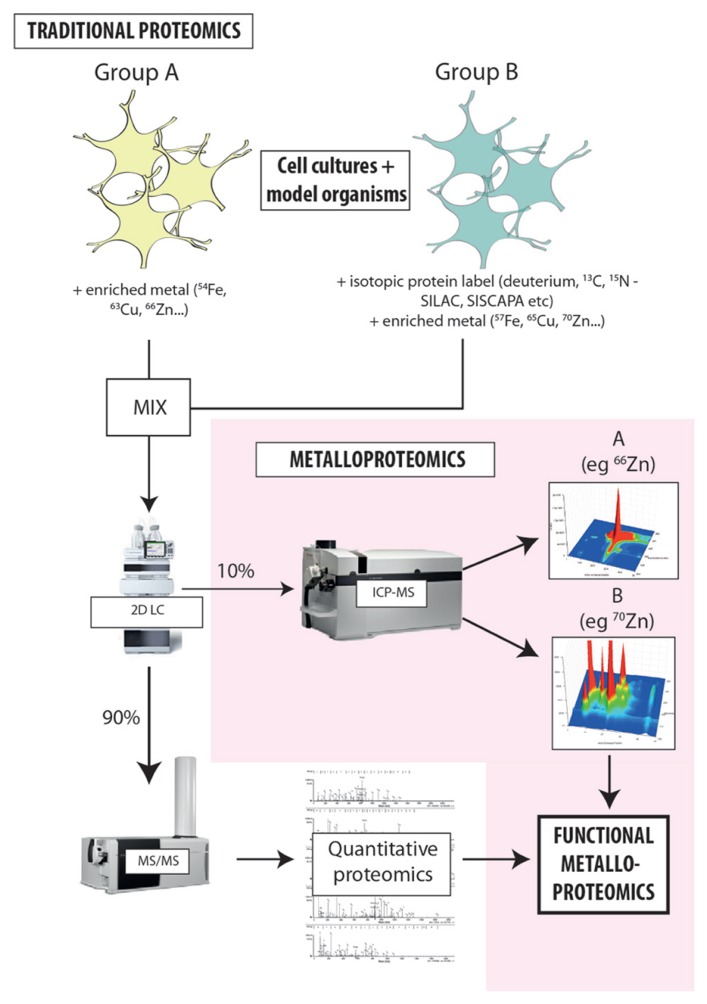
** Proposed workflow for integrated metalloproteomics.** Hyphenating native separation techniques to ICP-MS is the key to unlocking the secrets of metals and protein function. Rather than relying solely on bulk measures, directly associating metals with specific proteins provides new insight into how metals carry out biochemical processes in the cell. At the current state of our knowledge about the level of metalloproteins in biology the coupling of size exclusion to ICP-MS have the promise of being quantitative thus allowing the comparison of different samples and detail about the global or metalloprotein specific changes that occur. Despite SEC being a low resolution technique it can allow researchers to make educated guesses about the ID of proteins of interests based on their MW. Continued evolution of hyphenated LC-ICP-MS will only increase the arsenal of tools at our disposal to discover, identify, and characterize metalloproteins. The workflow developed in our laboratory adapts existing isotope labeling techniques used for proteomics [such as SILAC (stable isotope labeling by amino acids in cell culture) and SISCAPA (stable isotope standards and capture by anti-peptide antibodies)] to include the addition of isotopically enriched metal salts, providing new opportunities to probe the direct relationship metal cofactors have with protein function and allowing simultaneous analysis of both metals and proteins in individual experimental groups. Highly sensitive, isotope-specific ICP-MS detection is used to align metal distribution with quantitative proteomics, directly associating the presence of a protein species with a specific, metal-mediated function. This approach is extremely cost-effective, and can be seamlessly integrated into existing workflows with minimal disruption to the standard laboratory process.

The limitations to current metalloproteomics outlined above are not insurmountable; rather they simply require some lateral thinking regarding how we integrate modern analytical technology into systems biology. A variety of individual techniques can be combined for metalloproteomic experimental procedures, and these can generally be easily integrated into traditional proteomic workflows (**Figure 2**; Lancaster et al., 2011). By not reinventing the wheel, integration of atomic mass spectrometry (specifically ICP-MS) will allow for metal quantification and direct correlation between the presence of metal species and the function of associated proteins (Lelie et al., 2011; Alvarez et al., 2012). This comprehensive approach will greatly supplement the solely sequence information normally obtained through mass spectrometry independent of any other complementary technique.

Protein peaks identified from chromatographic separation can be correlated to the metal concentration peaks identified by on-line ICP-MS detection, allowing for the likely position of the metalloprotein to be determined, providing not only selectivity but also sensitivity (Gercken and Barnes, 1991; del Castillo Busto et al., 2005; Lopez-Avila et al., 2006; Manley et al., 2009). 2D separation offers a powerful tool for resolving complex mixtures of metalloproteins, and the multi-elemental capacity of ICP-MS produces a hyperspectral snapshot of metal–protein interactions in a single sample. Like traditional proteomics, this approach will use the vast arsenal of protein databases available to determine which of the proteins identified is most likely to exhibit metal–protein interaction. The 20,000+ proteins predicted in human samples will produce complex fractions highlighting the large dynamic range needed and requirement for fractionation.

Although there are inherent difficulties in investigating proteins in their native state, advances in mass spectrometry and analytical techniques are now making it possible to carry out investigations on intact proteins. Groundbreaking work from Joe Beckman’s laboratory has shown that using Fourier transform-ion cyclotron resonance MS it is possible to directly quantify and determine the metal status of a single protein from a specific cell type directly from tissue (Rhoads et al., 2011, 2013). This demonstrates that quantitative determination of both the amount of protein and the metal status can be achieved from biological tissue. Providing a new level of detail for a disease where it is clear that the metal status of a protein is key in the disease process (such as amyotrophic lateral sclerosis; Estévez et al., 1999), this technology is invaluable. The advent of top-down proteomics will be an increasingly valuable tool for metalloproteins identification.

## HOW DOES METALLOPROTEOMICS RELATE TO NEURODEGENERATION?

The brain is the most complex organ in the human body. Some of the proteins in the brain, just like the proteins in other tissues, require metal cofactors for function. Variations of the amount of metal that is present, or defects in the way a protein associates with a metal ion may cause disease states, particularly with regard to neurodegeneration.

The level at which metals are present within the brain is generally higher then the levels of the same metals in the rest of the body, and concentrations of metals in the brain is highly compartmentalized (Harrison et al., 1968; Hare et al., 2012b; Roberts et al., 2012). When metal homeostasis in these regions is altered, brain pathologies and increased oxidative stress is observed. As we age metals accumulate in the brain (Zecca et al., 2004) and this along with any other alterations in metal homeostasis can lead to neuronal damage, death, oxidative stress and may even cause misfolding and aggregation of proteins. However, it is important to point out that the accumulation of metals in tissues is not necessarily labile metals, but also metalloproteins. For example, it is estimated that there is less than one free Cu ion per cell (Rae et al., 1999). Thus an observed accumulation in bulk levels of Cu will have concomitant accumulation of Cu-metalloproteins. This shift in thinking about the general accumulation of metals to the change in functional metalloproteins is the basis of metalloproteomics. Bulk analysis only indicates a global change and still poses the question: do all metalloproteins change or are there specific targets? Our investigations suggest the latter.

In order to fully understand the roles that these metalloproteins play in age-related diseases it is first important to understand the role they play in the aging brain. Studies of trace elements and aging have shown consistent changes in trace elements such as a decrease in Rb and K and increase in metals Fe, Cu, and Co (Ehmann et al., 1984; Takahashi et al., 2001). The identification and characterization of metalloproteins is essential to understanding their functions and elucidating the specific disease pathways they are involved in, as well as assisting with the diagnosis of specific conditions (Gercken and Barnes, 1991; Muñiz et al., 2001; Lopez-Avila et al., 2006; El Balkhi et al., 2010). Characterized metalloproteins such as hemoglobin, transferrin, SOD, and ceruloplasmin are used in clinical laboratories as markers for specific disease states (Swart, 2013), such as anemia, inflammation (Ahluwalia, 1998), Down’s syndrome (Brooksbank and Balazs, 1984), and Wilson’s disease (Mak et al., 2008), respectively.

Metals have long been thought to play a role in pathophysiology of Alzheimer’s disease (AD) and Parkinson’s disease (PD). In AD, the concentration of three of the most abundant biochemically functional metals (Cu, Zn, and Fe) are altered in respect to their locations (i.e., metal redistribution in response to plaque formation). These metals are also thought to play a role in β-amyloid aggregation, which in turn will cause plaque formation, leading to the neurodegenerative effects seen in AD (reviewed in Roberts et al., 2012). The level of Cu in AD brain tissue is decreased, as it is hypothesized that Cu is removed from the tissue and associates itself with the senile plaques that are forming (Deibel et al., 1996; Hung et al., 2010). Zn is also thought to be associated with β-amyloid plaques (Bush et al., 1994), indicating that it has also been redistributed within the brain. Fe is also thought to be associated with plaques, but the overall change of Fe levels within the brain tissue surrounding fibril inclusions has yet to be described conclusively (Lovell et al., 1998; Schrag et al., 2011).

In PD, the levels of Fe and Zn have been suggested to be elevated, while the level of Cu may be decreased. Cu and Fe have been shown to interact with the protein alpha synuclein (Davies et al., 2011; Camponeschi et al., 2013), which is involved in proteinaceous Lewy body formation. Their interaction with alpha synuclein leads to crosslinking and protein aggregation (Barnham and Bush, 2008). Initial research into post-mortem changes in metals in PD dating back to the 1920s indicated that normal Fe became diminished from cells that are present in the globus pallidus and abnormal deposits of large quantities of Fe were found in globules in the same region of the brain (Lhermitte et al., 1924). Continued pursuit of elucidating the role of metals in PD pathogenesis has still only provided limited insight into the direct metal binding proteins involved, and limitations to analytical technology still present considerable problems for accurate quantification of metal changes (Hare et al., 2012a).

## CONCLUDING REMARKS

Perhaps the biggest impediment to the study of the metalloproteome has been technical limitations. However, the rapid advances in mass spectrometry and analytical chemistry in general over the past decade has helped, in some part, to overcome the shortfalls in the tools available to routinely study incredibly complex matrices. The ubiquitous nature of metal ions and metalloproteins coupled with our relatively limited knowledge about the metalloproteome presents an exciting frontier of new discoveries for the modern biochemist. As the continued growth in analytical technology finds new footholds in the life sciences, we can expect a transition from bulk analysis of trace elements to a detailed investigation of the metalloproteins. With this transition the interest in metalloproteomics will grow exponentially.

## Conflict of Interest Statement

Rudolf Grimm is an employee of Agilent Technologies. Dominic J. Hare and Blaine R. Roberts receive material support from Agilent. The remaining authors declare that the research was conducted in the absence of any commercial or financial relationships that could be construed as a potential conflict of interest.
